# Degree of exposure to interventions influences maternal and child dietary practices: Evidence from a large-scale multisectoral nutrition program

**DOI:** 10.1371/journal.pone.0221260

**Published:** 2019-08-26

**Authors:** Shalini Suresh, Anne Paxton, Bhim Kumari Pun, Min Raj Gyawali, Indra Dhoj Kshetri, Pooja Pandey Rana, Kenda Cunningham

**Affiliations:** 1 Department of Epidemiology, Columbia University Mailman School of Public Health, New York, NY, United States of America; 2 Helen Keller International, Kathmandu, Nepal; 3 CARE, Kathmandu, Nepal; 4 Digital Broadcast Initiative Equal Access, Kathmandu, Nepal; University of Dhaka, BANGLADESH

## Abstract

The prevalence of maternal and child malnutrition in Nepal is among the highest in the world, despite substantial reductions in the last few decades. One effort to combat this problem is *Suaahara* II (SII), a multi-sectoral program implemented in 42 of Nepal’s 77 districts to improve dietary diversity (DD) and reduce maternal and child undernutrition. Using cross-sectional data from SII’s 2017 annual monitoring survey, this study explores associations between exposure to SII and maternal and child DD. The study sample included 3635 mothers with at least one child under the age of five. We focused on three primary SII intervention platforms: interpersonal communication (IPC) by frontline workers, community mobilization (CM) via events, and mass media through a weekly radio program (Bhanchhin Aama); and also created an exposure scale to assess the dose-response relationship. DD was measured both as a continuous score and as a binary measure of meeting the recommended minimum dietary diversity of consuming foods from at least 5 of 10 food groups for mothers and at least 4 of 7 food groups for children. We used linear and logistic regression models, controlling for potentially confounding factors at the individual and household level. We found a positive association between any exposure to SII platforms and maternal DD scores (b = 0.09; p = 0.05), child (aged 2–5 years) DD scores (b = 0.11; p = 0.03), and mothers meeting minimum dietary diversity (OR = 1.16; p = 0.05). There were significant, positive associations between both IPC and CM events and meeting minimum DD (IPC: OR = 1.31, p = 0.05; CM: OR = 1.37; p<0.001) and also between CM events and DD scores (b = 0.14; p = 0.03) among mothers. We found significant, positive associations between mass media and meeting minimum DD (OR: 1.38; p = 0.04) among children aged 6–24 months and between mass media and DD scores (b = 0.15; p = 0.01) among children aged 2–5 years. We also found that exposure to all three platforms, versus fewer platforms, had the strongest association with maternal DD scores (b = 0.45; p = 0.01), child (aged 2–5 years) DD scores (b = 0.41; p<0.001) and mothers meeting MDD (OR = 2.33; p<0.001). These findings suggest that a multi-pronged intervention package is necessary to address poor maternal and child dietary practices and that the barriers to behavior change for maternal diets may differ from those for child diets. They also highlight the importance of IPC and CM for behavior change and as a pre-requisite to mass media programs being effective, particularly for maternal diets.

## Introduction

Although the prevalence of maternal and child undernutrition in Nepal has greatly reduced over the last two decades, the prevalence remains among the highest in the world [1]. The most recent Nepal Demographic and Health Survey (DHS) found that 17% of women of reproductive age (WRA) are underweight (body mass index <18.5) and 41% are anemic (hemoglobin <11 g/dl if pregnant and <12 g/dl if non-pregnant) [2, 3]. Among children under 5 years, 36% are stunted (height/length-for-age less than 2 standard deviations), 10% are wasted (weight-for-height/length less than 2 standard deviations) and 53% of children aged 6–59 months are anemic (hemoglobin <11 g/dl) [2, 3].

Infant and young child feeding (IYCF), however, has not improved in Nepal for the last several decades [3, 4]. Dietary diversity (DD) among children 6–23 months remains poor with only 47% meeting the child minimum dietary diversity (MDD) requirement of consuming foods from at least 4 of 7 food groups [1, 2, 4, 5]. Only about half of mothers with a child under 2 years of age meet MDD requirements of consuming foods from at least five of ten key food groups [2, 6]. Several cohort studies have found low DD scores among mothers in Nepal [7–9]. Maintaining adequate dietary practices in Nepal can be challenging due to widespread food insecurity, insufficient availability of and access to diverse foods, or simply staple-oriented diets, which compromise diverse dietary intake [10]. A lack of knowledge of appropriate practices, limited access to healthcare services and supplies, and poor availability of quality services often prevent women from adopting ideal health practices [11].

The Government of Nepal (GoN) is currently implementing the second phase (2018–2022) of its Multi-Sectoral Nutrition Plan (MSNP) with support from external development partners [12]. *Suaahara* II (SII), a five-year (2016–2021) USAID-funded multisectoral program that builds on an initial five years of implementation during phase one (2011–2016), aligns with and supports the GoN’s MSNP to reduce maternal and child undernutrition. Helen Keller International (HKI) leads the implementation of SII in partnership with six core implementing partners (CARE, Digital Broadcast Initiative Equal Access, Environment and Public Health Organization (ENPHO), FHI360, Vijaya Development Resource Center (VDRC) and Nepal Technical Assistance Group (NTAG), and local organizations in each SII district. With a target of reaching 1.5 million women and children across 42 of Nepal’s 77 districts, SII’s primary objective is to reduce undernutrition and anemia among children under 5 years of age and women of reproductive age (WRA) (15–49 years). SII’s multi-sectoral activities span: nutrition, health including family planning, water, sanitation and hygiene (WASH), agriculture/homestead food production (HFP), governance, and gender equity and social inclusion (GESI).

SII works with GoN frontline workers (FLWs) such as Female Community Health Volunteers (FCHVs), health workers, and agriculture/livestock extension workers. SII also has its own complementary FLW cadre including Field Supervisors (FS), Community Nutrition Facilitators (CNF), Community WASH Facilitators/ Triggerers, and Village Model Farmers (VMFs). In 2017, SII employed 600 FS to carry out household and community-level activities, in coordination with the nearly 27,725 FCHVs present in the SII districts. SII’s Social and Behavior Change Communication (SBCC) approach uses three main platforms to reach target beneficiaries: interpersonal communication (IPC), community mobilization via events (CM), and mass media. IPC activities include home visits made by SII FS to 1000-day households to provide one on one counselling and support. Community-level fairs and interactions, including food demonstrations and key life events to celebrate pregnancy, birth, and a child turning six months of age are examples of SII community events held to promote key nutrition and health behaviors. SII’s mass media approach primarily includes *Bhanchhin Aama* (BA), a weekly radio program complemented by a follow-up weekly call-in show.

It is important to understand which and how many of these interventions are necessary to achieve desired behavioral outcomes. A previous study using data from the first phase of *Suaahara* found that households exposed to *Suaahara* interventions had better knowledge related to child and maternal health and better sanitation practices, compared to households not exposed to the interventions [13]. Evidence shows that combining different interventions accelerates the rates at which child and maternal nutritional status improvements can occur when compared to what is achievable with each individual intervention alone [14]. However, there is a lack of sufficient research on the most effective combination of intervention platforms and, thus, a global call acknowledges the need for more solid evidence surrounding implementation, utilization and scaling up of diverse nutrition interventions [15].

This study examines the relationship between exposure to SII overall, exposure to each SII platform, and the degree of exposure to these platforms and maternal and child DD, using data from a cross-sectional survey conducted in 2017 as part of SII’s monitoring system. The purpose is to generate hypotheses regarding the effectiveness of different types of intervention platforms to start to fill evidence gaps on the additive nature of varying intervention platforms in complex large-scale programs.

## Methods

### Survey design and sampling

The data for this study was obtained from a representative, cross-sectional survey among households with children under 5 years of age conducted one year after the start of the SII program. New ERA, an external survey firm, carried out this first annual monitoring survey for SII between June 10 to September 10, 2017, the rainy season in Nepal. Multi-stage cluster sampling was conducted to select: 16 districts, 1 rural and 1 urban municipality per district (N = 32), 3 wards per municipality (N = 96), 2 sub-wards/clusters per ward (N = 192), and 19 households with a child under 5 years of age per cluster (N = 3648). The first four stages were conducted using probability proportion to size (PPS) techniques. For the fifth stage, households with a child under 5 years of age and his/her mother in residence were selected randomly from a full list of households within each cluster, created by the survey firm in the few days prior to the survey. In total, 3643 households with at least a mother and one child (her own) below the age of 5 years were included as study participants, and we had complete data on 3635 mothers, as 1 participant refused consent and 7 study participants consented but withdrew participation during the survey.

The household survey included questionnaires targeted to mothers, fathers or another male household head (or female, if no male available), and grandmothers living in the same household. Information on household socio-demographics and economics; knowledge and practices related to nutrition, health, family planning, agriculture/HFP, and WASH; empowerment; mental health; dietary intake for adults and the youngest child under 5 years of age; and exposure to SII messages, platforms, and FLWs.

### Variables

For this study, three exposure variables were created, one for each of the three main SII intervention platforms: IPC, CM, and mass media (*Bhanchhin Aama)*. A binary variable was created for each as follows:

1. IPC: met with any *Suaahara* FLW (field supervisor, village model farmer, or WASH triggerer) in the past six months; this does not include any government workers aligned with *Suaahara*, for example, FCHVs.
2. CM: ever participated in *Suaahara* community events such as food demonstrations, ideal family celebrations, key life events, WASH triggering sessions, Community Health Score Board (CHSB) interactions and special day, week and month celebrations, which may be done in collaboration with FCHVs but absent *Suaahara*, are not common FCHV activities.
3. Mass media: ever listened to the *Bhanchhin Aama* (BA) weekly radio program, designed aired and promoted by *Suaahara*

A scale (0–3) was also created to categorize participants based on the extent of their exposure to these SII platforms. We generated a categorical variable with four categories: zero, one, two, and three platforms.

Maternal and child DD indicators include DD scores and minimum dietary diversity (MDD). Dietary data was collected using a semi-quantitative 24-hour dietary recall method. Following the globally recommended women’s dietary diversity score, foods consumed by the mother were grouped into 10 food groups: 1) grains, white roots and tubers, and plantains; 2) pulses (beans, peas and lentils); 3) nuts and seeds; 4) dairy; 5) meat, poultry and fish; 6) eggs; 7) dark green leafy vegetables; 8) other vitamin A-rich vegetables and fruits; 9) other vegetables; 10) other fruits. From this, women’s DD scores (range of 0–10) were calculated as the total number of distinct food groups consumed and MDD was defined as consumption of foods from at least 5 of these 10 food groups [6].Foods consumed by the young child were grouped into 7 food groups: 1) milk, other than breast milk, and dairy such as cheese and yogurt; 2) grains, roots, and tubers; 3) vitamin A-rich vegetables and fruits; 4) other fruits and vegetables: 5) eggs; 6) meat, poultry and fish and 7) legumes and nuts. From this, child DD scores (range of 0–7) were calculated as the total number of distinct food groups consumed and MDD was defined as consumption of foods from at least 4 of these 7 food groups [5]. In this dataset, dietary diversity scores were normally distributed and thus, transformation was not necessary.

### Data analyses

Initial analysis comprised of descriptive analyses to generate means and standard deviations for continuous demographic variables such as age, years of education, number of children in the household, and DD scores. Additionally, descriptive analyses of the binary and categorical variables were performed to obtain percentage distributions of the sample population among the categories. To assess the differences in these variables between the mothers in the sample who were exposed to SII and those who weren’t, chi-square tests were conducted to generate p-values that indicated the significance of the differences.

Linear regression models were used to model the relationships between exposure to each platform and the continuous outcome variables—DD scores -, which had a normal distribution. Similarly, logistic regression models were used to model the relationships between exposure to each platform and the binary MDD outcome variables. All observations were independent of each other and minimum sample sizes for all regressions were met. To assess the dose-response relationship between the degree of exposure and the outcomes of interest, a logistic regression was used for all outcomes with the exposure variable being the scale of platform exposure, and the reference point being exposure to zero platforms. The outputs from the linear regressions provided beta values and p-values and the outputs from the logistic regressions provided beta values, odds ratios, and p-values. All of these statistical outputs were assessed to determine associations between the exposures and outcomes of interest.

All models were adjusted for potentially confounding factors identified a-priori including mothers’ age and education level, number of children under 5 years of age in the household, household socio-economic status, caste/ethnicity, agroecological zone, and urban/rural residency. Child regression models were also adjusted for child age, child gender and any child illness in two weeks prior to the survey were also added as potential confounders. Maternal age and education level were constructed as continuous variables with education level referring to total number of years of formal education received. The number of children under 5 years of age in the household and urban/rural residence were included as binary variables. Household socio-economic status was measured with the use of the Equity Tool, which uses ownership of key assets and quality of household structures (walls, roofs, and floors) to generate equity quintiles [16]. Caste/ethnicity was categorized into three groups: Brahmins/Chhetri (socially advantaged); social excluded groups (Dalit, Muslim, disadvantaged Janajati); and other groups (Gurung/Thakali, Newar, other non-Dalit Terai castes, others) [17, 18]. Agroecological zone was a categorical variable constructed to represent the three diverse geographical zones in Nepal: the *terai* (plains), hills and mountains, each of which vary in availability of diverse foods and have cultural and linguistic differences. Child age was constructed as a continuous variable referring to total number of completed months of age. Child gender was constructed as a binary variable with two categories of male and female. Any child illness was also constructed as a binary variable with one category referring to the child having any type of illness in the two weeks prior to the survey and the second category referring to the child not having any type of illness in the two weeks prior to the survey. Additionally, all regressions were adjusted for clustering at the smallest geographic unit of the survey (sub-ward).

### Ethics

Ethical approval for this survey was obtained from the Nepal Health Research Council (NHRC). Each respondent included in the survey provided written informed consent prior to beginning any interview, and the consent process was repeated after the completion of each module in the questionnaires, in order to continue the survey.

## Results

Detailed background characteristics of the survey sample are presented in Table 1. The mean age of all mothers was 26 years and they had an average of 6 years of formal schooling. About half of the survey sample identified as belonging to a socially excluded caste/ethnic group and almost three-quarters belonged to the three lowest equity quintiles. In this sample of mothers, their mean DD score was 4 out of 10 food groups and just over a third of the mothers met MDD recommendations of consuming foods from 5 out of 10 food groups (Table 1).

**Table 1:** Background characteristics of *Suaahara* II’s 2017 household-level monitoring survey sample.

Sample characteristics	All (N = 3635) Mean (SD)/ % (n)	Exposed (N = 1268) Mean (SD)/ % (n)	Unexposed (N = 2367) Mean (SD)/ % (n)	Significance of differences: *p-value*
Mothers' age (years; range: 15–49)	26.2 (5.5)	26.1 (5.4)	26.3 (5.6)	0.43
Mothers' education (years; range: 0–18)	6.1 (4.3)	7.1 (4.1)	5.6 (4.3)	0.00
Child age (months; range: 0–59)	24.7 (16.0)	24.8 (15.6)	24.7 (16.2)	0.87
Child sex: female	44.4% (1615)	44.7% (567)	44.3% (1048)	0.80
Number of children <5 years in household	1.2 (0.4)	1.2 (0.4)	1.2 (0.5)	0.86
**Caste**				0.00
Brahmin/Chhetri	39.4% (1431)	43.7% (554)	37.1% (877)	
Socially excluded	49.5% (1800)	48.6% (616)	50.0% (1184)	
Other	11.1% (404)	7.7% (98)	12.9% (306)	
**Equity quintile**				0.00
Quintile 1 (lowest)	21.7% (789)	23.3% (296)	20.8% (493)	
Quintile 2	28.6% (1041)	31.7% (402)	27.0% (639)	
Quintile 3	23.2% (842)	24.2% (307)	22.6% (535)	
Quintile 4	20.4% (740)	16.3% (207)	22.5% (533)	
Quintile 5 (highest)	6.1% (223)	4.4% (56)	7.1% (167)	
**Agroecological zone**				0.00
Terai	31.3% (1139)	14.4% (182)	40.4% (957)	
Hills	56.2% (2044)	69.5% (881)	49.1% (1163)	
Mountains	12.5% (452)	16.2% (205)	10.4% (247)	
Residence: rural area	50.0% (1816)	57.4% (728)	46.0% (1088)	0.00
Mothers’ dietary diversity score (range: 1–8)	4.1 (1.2)	4.3 (1.2)	4.0 (1.1)	0.00
Mothers’ minimum dietary diversity (5+ of 10FG)	35.5% (1292)	41.6% (528)	32.3% (764)	0.00
Child dietary diversity score (among >6m, N = 3157) (range: 0–7)	3.6 (1.1)	3.8 (1.1)	3.5 (1.1)	0.00
Child minimum dietary diversity (among >6m, N = 3157; 4+ of 7FG)	54.5% (1720)	60.8% (669)	51.1% (1051)	0.00

The significant differences found between individuals exposed versus not exposed to any SII platforms were that the exposed women had, on average, 1.5 years more of schooling. A higher percentage of the exposed women were also of a higher caste/ethnicity group, fell into the upper equity quintiles, were from the hills, and resided in rural areas. In addition, outcomes of diet diversity scores and meeting minimum dietary diversity were significantly different between exposed and unexposed mothers and children, based on chi-squared tests (p<0.001).

After one year of SII implementation, slightly over one-third of the mothers in the sample had been exposed to any of the three SII platforms (Table 2). Exposure was highest to mass media (listening to BA), followed by participation in SII-led CM events and lastly, one-on-one IPC with SII FLWs. Of the 10% of mothers who were exposed to IPC, two-thirds had one FLW interaction while one-third had two or more. Almost 90% of those who ever participated in a CM event, participated in exactly one community event. Among those who had ever listened to BA, almost two-thirds listened to it multiple times in a month. The exposure scale revealed that the majority (65%) of mothers had no exposure to SII.

**Table 2:** Exposure to *Suaahara* II platforms among mothers.

Exposure	Mothers of children under 5 (N = 3635)

	% (n)
**Any exposure to each platform**	
Interpersonal communication with SII frontline worker (in last 6 months)	10.8% (393)
Community mobilization (ever participated in any event)	13.0% (473)
Mass media (ever listened to *Bhanchhin Aama*)	21.7% (790)
Any of the above	34.9% (1268)
**Degree of exposure to each platform**	
**Number of FLW interactions (in last 6 months)**	
0	89.6% (3258)
1	6.9% (249)
2 or more	3.5% (128)
**Number of community events (ever)**	
0	87.0% (3162)
1	11.5% (419)
2 or more	1.5% (54)
**Frequency of listening to *Bhanchhin Aama***	
Never	78.3% (2845)
Less than once a month	6.1% (221)
Once a month	1.8% (66)
Multiple times in a month	13.8% (503)
**Scale of exposure**	
0	65.1% (2367)
1	25.8% (939)
2	7.4% (270)
3	1.6% (59)

We found that exposure to any SII platform was positively associated with mothers’ DD scores (b = 0.09; 95% CI: 0.00, 0.17; p = 0.05) and meeting MDD (OR: 1.16; 95% CI: 1.00, 1.36; p = 0.05) (Table 3). Specifically, mothers who had any IPC by interacting with a SII FLW in the last 6 months had 1.31 times the odds of meeting MDD than those who had no IPC (95% CI: 1.00, 1.72; p = 0.05). Similarly, the mothers who had ever participated in a SII CM event had 1.37 times the odds of meeting MDD, in comparison to those who had never participated (95% CI: 1.11, 1.70; p<0.001). Maternal DD scores were also significantly higher among mothers who had ever participated in a CM event by 0.14 units, compared to those who had never participated (95% CI: 0.02, 0.26; p = 0.03). There were no significant associations between listening to BA and maternal DD.

**Table 3:** Multivariate associations* of maternal exposure to *Suaahara* II intervention platforms and maternal dietary diversity.

	Dietary Diversity Score (N = 3635)		Minimum Dietary Diversity (N = 3635)	
	Beta (95% CI)	*p*	OR (95% CI)	*p*
**Any exposure (IPC = interpersonal communication; CM = community mobilization events; MM = mass media)**				
**IPC**	0.15 (-0.01, 0.31)	0.07	1.31 (1.00, 1.72)	0.05
**CM**	0.14 (0.02, 0.26)	0.03	1.37 (1.11, 1.70)	<0.001
**MM**	0.07 (-0.02, 0.17)	0.14	1.08 (0.91, 1.28)	0.38
**Any**	0.09 (0.00, 0.17)	0.05	1.16 (1.00, 1.36)	0.05
**Scale (reference group: 0)**				
**1**	0.04 (-0.05, 0.14)	0.38	1.08 (0.92, 1.27)	0.36
**2**	0.18 (0.00, 0.36)	0.05	1.34 (0.99, 1.83)	0.06
**3**	0.45 (0.14, 0.76)	<0.001	2.31 (1.46, 3.65)	<0.001

* Adjusted for: clustering and mother’s age and years of education, number of children under 5 living in household, socio-economic status, caste/ethnicity, agro-ecological zone, urban/rural residency.

We also found a positive trend of improved maternal DD with increasing exposure to SII platforms. Exposure to two of the three platforms was correlated with higher DD (b = 0.18; 95% CI: 0.00, 0.36; p = 0.05) for mothers and exposure to all three SII platforms had the largest association. Mothers exposed to all three platforms had DD scores that were 0.45 units higher than mothers who were exposed to none (95% CI: 0.14, 0.76; p<0.001) and they were more than twice as likely to meet MDD than mothers with exposure to none (95% CI: 1.46, 3.65; p<0.001) (Table 3).

We explored the associations between exposure to SII and child dietary indicators by splitting the sample between children 6–24 months of age and children 2 to 5 years of age because children 6–24 months of age remain within the critical 1000-day growth period, are still breastfeeding, and are also SII’s primary target population (Table 4). We found that exposure to any SII platform was positively associated with child DD scores (b = 0.11; 95% CI: 0.01, 0.21; p = 0.03) for the older children, but not with whether they met MDD. There was no association for either dietary indicator for the younger children. We found associations, however, between exposure to mass media and MDD scores for younger children (b = 0.32; 95% CI: 0.01, 0.63; p = 0.04) and DD scores for older children (b = 0.15; 95% CI: 0.04, 0.26; p = 0.01), We did not find any association between either IPC or participation in CM events and child DD.

**Table 4:** Multivariate associations* of maternal exposure to *Suaahara* II intervention platforms and child dietary diversity.

	Dietary Diversity Score: 6–23.9m (N = 1383)		Minimum Dietary Diversity: 6–23.9m (N = 1383)		Dietary Diversity Score: 24-59m (N = 1774)		Minimum Dietary Diversity: 24-59m (N = 1774)	
	Beta (95% CI)	*p*	OR (95% CI)	*p*	Beta (95% CI)	*p*	OR (95% CI)	*p*
**IPC**	0.01 (-0.20, 0.22)	0.91	1.05 (0.68, 1.63)	0.82	0.11 (-0.07, 0.28)	0.24	1.12 (0.79, 1.59)	0.52
**CM**	-0.06 (-0.26, 0.14)	0.58	0.82(0.54, 1.24)	0.36	0.11 (-0.03, 0.26)	0.12	1.09 (0.83, 1.44)	0.53
**MM**	0.11 (-0.05, 0.27)	0.18	1.38 (1.01, 1.88)	0.04	0.15 (0.04, 0.26)	0.01	1.18 (0.95, 1.45)	0.14
**Any**	0.09 (-0.04, 0.22)	0.19	1.24 (0.95, 1.61)	0.12	0.11 (0.01, 0.21)	0.03	1.14 (0.92, 1.41)	0.23
**1**	0.11 (-0.04, 0.26)	0.15	1.27 (0.95, 1.71)	0.11	0.07 (-0.04, 0.17)	0.20	1.06 (0.84, 1.34)	0.60
**2**	0.06 (-0.22, 0.35)	0.67	1.20 (0.66, 2.17)	0.56	0.19 (0.03, 0.36)	0.02	1.44 (0.98, 2.12)	0.07
**3**	-0.27 (-0.69, 0.15)	0.20	0.61 (0.22, 1.72)	0.35	0.41 (0.16, 0.67)	<0.001	1.39 (0.68, 2.88)	0.37

Adjusted for: clustering and child age and gender, mother’s age and years of education, number of children under 5 living in household, socio-economic status, caste/ethnicity, agro-ecological zone, urban/rural residency, any child illness in two weeks prior to the survey.

We also found a positive trend with increasing exposure to SII platforms for older children’s DD scores: those with exposure to two of the three platforms had higher DD than those exposed to one (b = 0.19; 95% CI: 0.03, 0.36; p = 0.02) and exposure to all three SII platforms had the largest association (b = 0.41; 95% CI: 0.16, 0.67; p<0.001). Similarly, children exposed to two or three platforms were 1.4 times likely to reach MDD, but this was only significant for those exposed to two platforms (OR: 1.4, 95% CI: 0.98, 2.12; p = 0.07).

## Discussion

This study investigates associations between exposure to *Suaahara II* (SII), including three main SBCC intervention platforms–interpersonal communication, community events, and mass media–and maternal and child dietary diversity. We found that interpersonal communication, specifically interaction with SII frontline workers, and participation in SII-organized community mobilization events had positive, significant associations with maternal dietary diversity, whereas the only association with child dietary diversity was exposure to mass media. We also found that the more platforms a mother was exposed to, the stronger the positive association with maternal dietary diversity was: mothers who were exposed to 2, versus 1, platform, had a more than twofold higher DD score. A similar finding was true for children, especially those 2 to 5 years of age. This suggests that exposure to multiple intervention platforms, rather than just one type of intervention, may be important for driving improvements in dietary diversity.

The overall exposure to these three SII platforms is seemingly low, although expected after only one year of implementation particularly in such an at-scale program. There is significant lag time to contract community-based NGOs, set up offices across the country, hire and train FLWs and finally, implement at the household and community levels. Furthermore, given the ratio of SII staff to the target households (625 FS: 750,000 households), we would expect the exposure levels to be low after year one, but also expect this to improve over the program duration. SII also introduced a new line of FLWs in the second program year, about 800 Community Nutrition Facilitators (CNFs), to increase coverage of program interventions.

We further explored the dietary data by comparing the consumption of specific food groups among mothers and children who were exposed to any SII interventions versus those who were not (results not shown). The key differences found were that mothers who were exposed to SII consumed dairy and dark green leafy vegetables significantly more than mothers who weren’t exposed. In addition, children of mothers who were exposed to SII consumed significantly higher amounts of dairy, eggs and Vitamin-A rich fruits and vegetables. SII promotes the consumption of both dark green Vitamin A rich vegetables and animal source foods and thus this finding is consistent with the intervention theory of change. Other SII-promoted foods, such as meat, are more expensive and are not a part of the daily Nepali diet, suggesting that promotion of the consumption of dairy, eggs and dark green leafy vegetables may be a feasible way of improving dietary diversity among mothers and children in Nepal.

Since SII activities are integrated and are carried out in villages covering more than half the country, dissecting which activity covered which topic (agriculture, WASH, health, etc.) is challenging. However, to understand why IPC and CM, but not mass media, were associated with maternal dietary diversity, we looked more closely at the content of these different SBCC platforms. First, during IPC, some of the specific counselling messages given by SII during home visits vary depending on stage in the 1000-day period, such as ANC and PNC, but maternal and child diets are always a focus. Dietary diversity is also a significant focus of FLWs during nearly all community events. Food demonstrations are solely concentrated on improving diets, but key life celebrations held for each mother during her pregnancy, after delivery and when her child turns 6 months of age also revolve around food: the mother is given a nutrition-related gift basket from her community which often includes nutritious locally grown foods to discuss the importance of maternal and child dietary diversity. However, only 3 of the 35 BA episodes aired during the first year of SII covered dietary diversity. This may explain why IPC and CM events, and not BA, were associated with maternal diets.

For children, only mass media had a significant association with dietary diversity. This finding may reflect on the fact that the radio program focused more on child, rather than adult, diet practices. The seemingly contradictory results around the associations between different interventions for mothers and children reveal that the determinants of maternal and child dietary practices may differ, with the same platforms having different effects on the uptake of appropriate dietary practices. This finding emphasizes that a combination of approaches may be necessary to bring about sustained positive change in household dietary practices.

Exposure to all three platforms had the strongest, positive association with dietary outcomes for mothers and a similar, although less consistent and significant pattern, emerged for child dietary outcomes as well. This result that a combination of platforms had a stronger association with nutrition practices confirms findings from prior studies conducted in Nepal [13], Bangladesh [14], and Vietnam [19]. Descriptive analyses showed that those who had ever listened to BA seemed to listen to it quite often, since almost two-thirds of these mothers reported listening to it multiple times in a month. Hence, reception to the radio program is positive and encouraging, and it suggests that there is potential for high levels of uptake. The influence of mass media through platforms such as televisions and radios on improving health practices have also been widely recognized to have correlations with improved maternal health knowledge and practices [19–21]. This implies that it is imperative to expand the reach of this platform and optimize its potential for impact on maternal nutrition and health practices, but that mass media is an add-on intervention that cannot replace interpersonal communication and other face to face engagements with beneficiaries.

A major limitation of this study is that the dataset is cross-sectional and thus, we cannot infer causality from any of the significant associations identified in these results. The inability to remove potential residual confounding may have resulted in some bias which must also be considered in the interpretation of the results. We considered categorizing the exposure platforms based on intensity of exposure, for example number of FLW interactions, and also assessing the relationship between every possible combination of platforms and our outcomes. However, the sample size for each sub-group was too small and would not have allowed us to generate robust and reliable regression results. Finally, collinearity of exposure platforms is an issue. The three exposure platforms were significantly associated, which is expected since each platform would be promoted among the other platform activities–for example, during home visits FLWs promote participation in community events and that mothers listen to the BA radio program. However, we could not adjust for this in our regressions as each exposure platform would be on the causal pathway between the other exposure platforms and the outcomes and thus the findings for each would be underestimated. Additionally, we were unable to disentangle joint FCHV-SII interventions, because FCHVs are government workers and are not solely SII FLWs. Despite these limitations, the findings of this study are informative and can be used to guide decisions on where to invest programmatically, particularly for the remaining three years of SII.

Additional analysis of this data and future rounds of this monitoring survey are needed to answer questions including how these platforms relate to other maternal and child nutrition and health outcomes, particularly as exposure to the platforms increases. Qualitative studies investigating the differences between exposure platforms in terms of reach and uptake would be useful to understand why some platforms work better and how the radio program could be improved to have a stronger impact. More rigorous studies such as a randomized controlled trial would be necessary to minimize bias and establish causal relationships between the platforms and specific practices.

## Conclusion

The findings of this study will have important implications on future implementation of SII and other similar large-scale nutrition programs. There needs to be a strong focus on strengthening the quality and reach of individual intervention platforms, specifically FLW interactions and community events, given the demonstrated strong individual associations with dietary outcomes. Finally, the effectiveness of the combinations of platforms suggested by the increasing association of exposure with positive diet outcomes highlight the need for nutrition programs and government policies to adopt a multi-platform model to have the highest potential for reach and positive impact on maternal and child nutrition practices.

## Supporting information

S1 Data Anonymized data. (XLS)
